# Histopathological Risk Features in Primary and Secondary Enucleation for Group D Retinoblastoma: A Clinicopathological Correlation Study

**DOI:** 10.7759/cureus.102113

**Published:** 2026-01-22

**Authors:** Khawaja Muhammad Ammar Ali Javed, Anum Javed, Usman Vayani, Muhammad Hanif Chatni

**Affiliations:** 1 Academic Unit of Ophthalmology, University of Birmingham, Birmingham, GBR; 2 Ophthalmology, Al Ibrahim Eye Hospital, Karachi, PAK; 3 Ophthalmology, Patel Hospital, Karachi, PAK

**Keywords:** clinicopathological correlation, histopathology, intraocular tumours, retinoblastoma group d, surgical enucleation

## Abstract

Background and objective

Group D retinoblastoma (RB) represents advanced intraocular disease with a substantial risk of treatment failure. Although globe-sparing therapies have improved ocular salvage rates, there is limited knowledge regarding the histopathological risk profile of enucleated Group D eyes, particularly when enucleation is performed after failed conservative treatment. This study aimed to evaluate the presence of high-risk histopathological features in Group D eyes undergoing primary versus secondary enucleation.

Methods

This study was designed as a focused secondary histopathological analysis of enucleated eyes from a previously reported cohort of Group D RB patients managed at a tertiary care center. Eight eyes underwent enucleation: four (50%) underwent primary enucleation due to advanced disease at presentation, and four (50%) underwent secondary enucleation following the failure of globe-sparing therapy. All specimens were examined for high-risk histopathological features, including post-laminar optic nerve invasion, optic nerve cut-end involvement, massive choroidal invasion, and scleral invasion. Fisher’s exact test was used, and odds ratios (OR) with confidence intervals (CI) were calculated to explore differences between the groups.

Results

High-risk histopathological features were identified in four of eight eyes (50%). One of the four eyes that underwent primary enucleation (25%) demonstrated high-risk features, compared with three of the four eyes that underwent secondary enucleation (75%). Fisher’s exact test showed no statistically significant association between enucleation timing and high-risk pathology (p = 0.49). The OR indicated lower odds of high-risk pathology with primary enucleation (OR: 0.11; 95% CI: 0.005-2.73), although the confidence intervals were wide due to the small sample size.

Conclusions

High-risk histopathological features were more frequently observed in Group D RB eyes undergoing secondary enucleation after failed globe-sparing therapy. These findings suggest that delaying enucleation may allow the progression of microscopic disease that is not clinically apparent and highlight the importance of timely enucleation in selected advanced cases. This clinicopathological correlation complements reported clinical outcomes and supports careful risk assessment when considering globe-sparing approaches in Group D disease.

## Introduction

Retinoblastoma (RB) is the most common primary intraocular malignancy of childhood, with an estimated global incidence of one case per 15,000 to 20,000 live births [1]. While survival exceeds 95% in high-income countries, outcomes remain significantly poorer in many low- and middle-income countries (LMICs) due to delayed presentation, limited access to specialized care, and a higher proportion of children presenting with advanced intraocular disease [2-4]. Beyond survival, contemporary management increasingly emphasizes ocular salvage and visual rehabilitation, particularly with the advent of globe-sparing techniques such as systemic chemotherapy, intra-arterial chemotherapy (IAC), and intravitreal chemotherapy (IViC).

The International Classification of Retinoblastoma (ICRB) provides a widely accepted framework for staging intraocular disease, stratifying cases from Group A to Group E based on tumor size, location, and seeding characteristics [5]. Group D RB represents advanced intraocular disease, characterized by diffuse vitreous or subretinal seeding, large tumor burden, or extensive retinal detachment. Historically, the majority of Group D eyes underwent primary enucleation due to low salvage rates; however, modern multimodal approaches combining systemic chemotherapy, intra-arterial chemotherapy (IAC), intravitreal chemotherapy (IViC), and focal consolidation have improved globe salvage outcomes in selected patients [6-9]. Despite these advances, treatment failure remains common, and a substantial proportion of Group D eyes still eventually require enucleation.

Histopathological examination of enucleated eyes remains central to prognostication and postoperative management. High-risk histopathological features, particularly post-laminar optic nerve invasion, optic nerve cut-end involvement, massive choroidal invasion (>3 mm), and scleral infiltration, are strongly associated with an increased risk of extraocular spread and metastatic death if untreated [10-13]. Identification of these features informs the need for adjuvant systemic chemotherapy and influences the intensity of oncologic follow-up. The recognition of microscopic disease is especially important in LMIC settings, where delayed diagnosis and treatment interruptions are more common.

In the modern treatment era, an increasing number of enucleations are performed secondarily after the failure of globe-sparing therapy. Several reports have raised concerns that delaying enucleation in advanced eyes may allow progression of occult microscopic disease, even when partial clinical regression is observed [14-16]. However, relatively few studies have compared the histopathological risk profiles of eyes undergoing primary versus secondary enucleation, and there is a particular paucity of data focusing exclusively on Group D eyes, which represent an inherently high-risk and heterogeneous subgroup [17].

This study aims to address this gap by evaluating the prevalence and pattern of high-risk histopathological features in Group D RB eyes undergoing either primary or secondary enucleation at a tertiary care center in Pakistan. By correlating the timing of enucleation with pathological outcomes, this clinicopathological analysis complements previously reported clinical findings and provides valuable evidence to inform decision-making regarding timely enucleation in advanced intraocular RB.

## Materials and methods

Study design and setting

This retrospective clinicopathological study was conducted at a tertiary care ophthalmology center in Karachi, Pakistan, between 25 April 2013 and 1 December 2022. The analysis focused on the histopathological characteristics of enucleated eyes from a previously reported cohort of patients with Group D RB managed at the same institution [18]. The present work evaluates pathological risk features that were not assessed in the original outcomes study, providing additional clinicopathological correlation.

Ethical approval

This study was performed under institutional research approval granted by the Patel Hospital Ethical Review Committee (reference No. 110). The dataset analyzed here was obtained as part of this previously approved project. No additional patient recruitment or interventions were undertaken. All procedures adhered to institutional guidelines and the tenets of the Declaration of Helsinki, with strict maintenance of patient confidentiality.

Study population and patient selection

Between 25 April 2013 and 1 December 2022, 170 patients were referred to the Ophthalmology Department at Patel Hospital with a diagnosis of RB. Group D RB was identified in 28 patients. Of these, 22 patients had bilateral disease with Group D involvement in at least one eye, one patient had bilateral Group D disease, and six patients had unilateral Group D disease. Nine patients (32%) were excluded because they were evaluated only for a second opinion or were lost to follow-up. Consequently, 19 patients with Group D RB were included in the original clinical cohort [18]. From this cohort, eight eyes (8/19; 42%) ultimately underwent enucleation and formed the basis of the present histopathological analysis.

**Figure 1:**
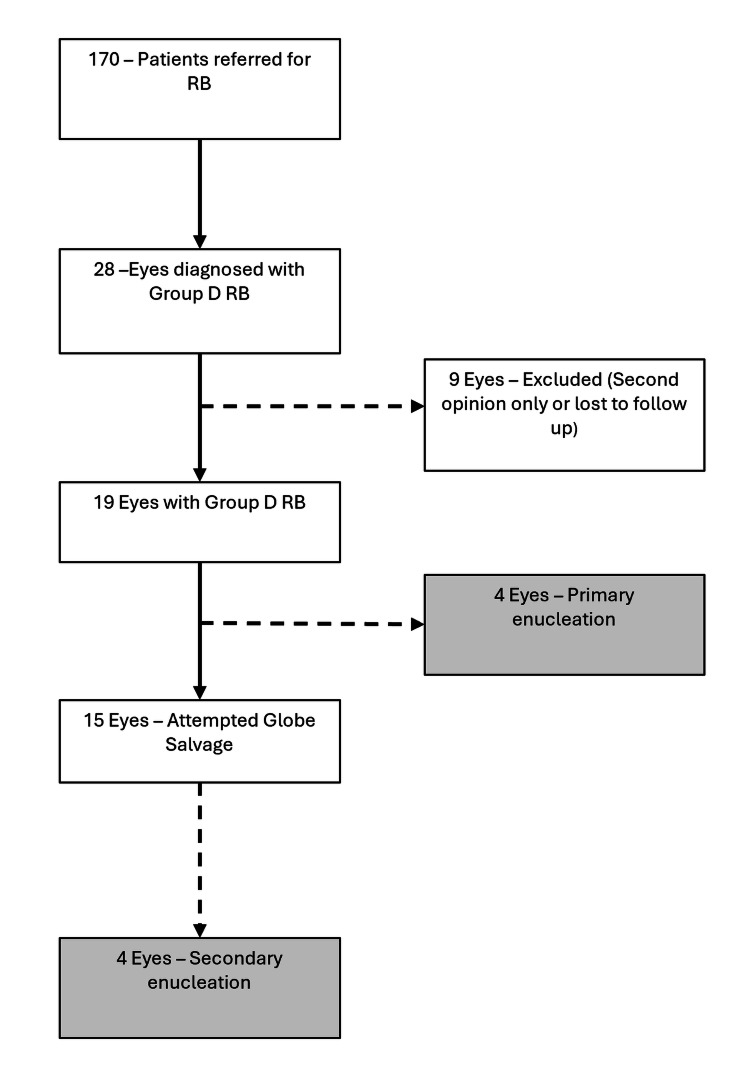
Cohort flow diagram depicting the selection of enucleated Group D RB eyes included in the histopathological analysis Between 25 April 2013 and 1 December 2022, 170 patients were referred to Patel Hospital for RB. Group D RB was identified in 28 patients; nine were excluded due to second-opinion evaluation only or loss to follow-up. Nineteen patients (19 Group D eyes) constituted the original clinical cohort. Of these, four eyes underwent primary enucleation at presentation, while 15 eyes underwent attempted globe-sparing therapy; four of these subsequently required secondary enucleation and were included in the present histopathological comparison RB: retinoblastoma

Group D classification was assigned based on established ICRB criteria [5]. Of the enucleated eyes, four eyes (50%) underwent primary enucleation due to advanced disease at presentation, while the remaining four eyes (50%) underwent secondary enucleation following documented failure of globe-sparing therapy, which included combinations of systemic chemotherapy, focal therapy, and intravitreal chemotherapy where indicated [18]. The patient selection process is summarized in Figure 1.

Histopathological processing and evaluation

All enucleated globes were fixed in 10% neutral-buffered formalin and processed using standard ophthalmic pathology techniques. Serial sections were stained with hematoxylin and eosin and examined by an experienced ocular pathologist. High-risk histopathological features were assessed in accordance with internationally accepted RB pathology guidelines and included (i) post-laminar optic nerve invasion, (ii) optic nerve cut-end involvement, (iii) massive choroidal invasion (>3 mm), and (iv) scleral invasion. Other pathological findings, such as anterior chamber involvement, iris or ciliary body infiltration, vitreous tumor infiltration, and degree of differentiation, were recorded but were not classified as high-risk for this analysis.

Outcome measures

The primary outcome was the presence of any high-risk histopathological feature. Secondary outcomes included the frequency of individual high-risk features and descriptive comparison of pathological profiles between eyes undergoing primary versus secondary enucleation.

Data analysis

Given the small sample size and the rare nature of this disease subgroup, data were analyzed descriptively. Categorical variables were summarized as frequencies and percentages. To explore differences between primary and secondary enucleation groups, Fisher’s exact test was used. Odds ratios (ORs) and 95% confidence intervals (CIs) were calculated to estimate the relative likelihood of high-risk pathology between groups. Statistical findings were interpreted cautiously, emphasizing effect size and clinical relevance rather than statistical significance alone. All analyses were performed using IBM SPSS Statistics for Windows, Version 26 (IBM Corp., Armonk, NY).

## Results

Enucleated cohort characteristics

Eight eyes clinically classified as Group D RB underwent enucleation during the study period. Four eyes (50%) underwent primary enucleation due to advanced disease at initial presentation, whereas the remaining four eyes (50%) underwent secondary enucleation following documented failure of globe-sparing therapy. Baseline clinical characteristics for both groups are summarized in Table 1. All specimens were successfully processed for histopathological evaluation without technical limitations.

**Table 1:** Baseline characteristics of enucleated Group D retinoblastoma eyes Baseline demographic and clinical features of the eight Group D eyes included in the study.

Variable	Primary enucleation (n = 4)	Secondary enucleation (n = 4)
Age at presentation, months, mean	14.8	15.1
Laterality – unilateral, n (%)	4 (100%)	3 (75%)
Laterality – bilateral, n (%)	0 (0%)	1 (25%)
Presenting symptom: leukocoria, n (%)	4 (100%)	4 (100%)
Prior globe-sparing therapy, n (%)	0 (0%)	4 (100%)
Intravitreal chemotherapy administered, n (%)	0 (0%)	3 (75%)
Age at enucleation, months, mean	14.9	27.3

Overall prevalence of high-risk histopathology

High-risk histopathological features were identified in four of the eight enucleated eyes (50%). Among eyes undergoing primary enucleation, one of four specimens (25%) demonstrated a high-risk feature. This eye exhibited post-laminar optic nerve invasion with associated anterior segment involvement, although no other high-risk elements were present. In contrast, three of the four eyes (75%) that underwent secondary enucleation demonstrated high-risk pathology. These eyes showed a broader and more advanced spectrum of microscopic invasion, including post-laminar optic nerve invasion, optic nerve cut-end involvement, massive choroidal invasion exceeding 3 mm, and, in one case, scleral infiltration. One specimen enucleated secondarily exhibited multiple high-risk features simultaneously, with extensive posterior invasion and deep choroidal involvement. The distribution of high-risk features across groups is detailed in Table 2.

**Table 2:** High-risk histopathological features and statistical comparison between primary and secondary enucleation Comparison of high-risk histopathological features between primary and secondary enucleation in Group D retinoblastoma. Only the composite outcome (“any high-risk feature”) was subjected to statistical testing due to sample size constraints ON: optic nerve; OR: odds ratio; CI: confidence interval

Feature	Primary enucleation (n = 4), n (%)	Secondary enucleation (n = 4), n (%)	OR (95% CI)	P-value
Post-laminar ON invasion	1 (25%)	2 (50%)	—	—
ON cut-end involvement	1 (25%)	2 (50%)	—	—
Massive choroidal invasion (>3 mm)	1 (25%)	1 (25%)	—	—
Scleral invasion	0 (0%)	1 (25%)	—	—
Any high-risk feature	1 (25%)	3 (75%)	0.11 (0.005–2.73)	0.49

Comparison of primary and secondary enucleation

High-risk pathological features were more frequently encountered in eyes enucleated after failed conservative therapy compared with eyes undergoing primary enucleation. Fisher’s exact test did not demonstrate a statistically significant association between enucleation timing and the presence of high-risk pathology (p = 0.49). The calculated OR indicated a lower likelihood of high-risk histopathology in eyes undergoing primary enucleation (OR: 0.11; 95% CI: 0.005-2.73), although the wide CI reflects the small sample size and the rarity of this disease subtype. A summary of statistical comparisons is presented in Table 2.

Patterns of individual pathological features

Evaluation of individual histopathological features revealed observable differences between the two groups. Post-laminar optic nerve invasion was identified in one primary (25%) and two secondary (50%) enucleations, while optic nerve cut-end involvement was likewise present in one primary (25%) and two secondary (50%) eyes. Massive choroidal invasion was detected in one eye (25%) from each group, although the secondarily enucleated specimen displayed a more extensive pattern of infiltration. Scleral invasion was observed exclusively in the secondary enucleation group, consistent with more advanced extraocular extension risk in eyes requiring delayed removal. Anterior segment involvement occurred in one primary (25%) and one secondary (25%) specimen but was not classified as a high-risk feature for the purposes of this analysis.

## Discussion

In this clinicopathological analysis of enucleated Group D RB eyes, we observed a higher prevalence and broader spectrum of high-risk histopathological features in eyes undergoing secondary enucleation compared with those treated by primary enucleation. Although the sample size is small, reflecting the rarity of this specific disease subset, the observed pattern is biologically plausible and aligns with an expanding body of clinicopathological literature. Multiple studies have reported that eyes requiring secondary enucleation after failed conservative therapy frequently harbor advanced posterior segment invasion despite appearing clinically controlled at earlier stages [14-17,19]. Collectively, these findings support the concept that delayed enucleation following unsuccessful globe-sparing therapy may permit progression of occult microscopic disease, even when clinical examination suggests partial response.

The overall frequency of high-risk histopathological features in this cohort (50%) is comparable to rates reported in other series examining enucleated advanced intraocular RB, where high-risk features have been identified in approximately 30-60% of cases depending on stage, treatment era, and geographic setting [8,10-12,17]. The substantially higher proportion of high-risk pathology observed among secondarily enucleated eyes (75%) compared with primarily enucleated eyes (25%) mirrors trends described in larger cohorts, in which delayed or secondary enucleation has been associated with increased risks of optic nerve and massive choroidal invasion [14-16,17]. Previous studies have reported that, despite significant advances in globe-sparing strategies, a subset of eyes ultimately requiring enucleation has demonstrated pathological invasion that was not clinically apparent, highlighting the limitations of clinical assessment alone in advanced disease [14,15,17]. Similarly, Chantada et al. demonstrated that delays in definitive local control were associated with increased risk of extraocular relapse, reinforcing the oncologic importance of timely enucleation in selected cases [11].

The predominance of optic nerve invasion and deep choroidal infiltration in secondarily enucleated eyes in the present study suggests that microscopic disease progression may occur during attempted globe salvage. This observation is consistent with prior pathological studies, which have shown that high-risk invasion can persist or progress despite apparent clinical regression of intraocular tumors or vitreous seeds [9,11-13,19]. Importantly, histopathology represents a terminal assessment and cannot distinguish between features present at diagnosis and those that developed during treatment; however, the consistent overrepresentation of high-risk features in secondarily enucleated eyes across multiple studies suggests that prolonged salvage attempts may increase the likelihood of identifying such features at the time of enucleation.

These findings are particularly relevant in LMIC settings, where delayed presentation, interruptions in therapy, and limited access to specialised treatment modalities remain common [2-4,18]. In such contexts, the risks associated with prolonged globe-sparing therapy may be amplified. Although IAC and IViC have improved local control rates, particularly for vitreous seeding [9,20], these modalities do not reliably prevent posterior segment invasion in all cases. Regression of vitreous seeds or tumor bulk does not equate to complete histopathological sterilisation, a discrepancy that has been emphasized in pathological correlation studies from both high- and low-resource settings [10,15,17,19]. Munier et al. highlighted the need for vigilant monitoring and timely conversion to enucleation when treatment resistance becomes evident, a principle that is supported by the pathological patterns observed in the present cohort [9].

The findings of this study reinforce the long-standing principle that enucleation, when performed at an appropriate time, remains a sight- and life-preserving intervention in selected cases of advanced RB. Children presenting with extensive tumor burden, recurrent vitreous seeding, or persistent retinal detachment may be at particular risk of harboring high-risk pathological features despite multiple rounds of conservative therapy. As emphasized by the International Retinoblastoma Staging Working Group, early identification of high-risk histopathological features is critical, as these parameters directly inform decisions regarding adjuvant systemic chemotherapy and long-term oncologic surveillance [10]. The higher burden and complexity of high-risk features observed in secondarily enucleated eyes in this study support maintaining a low threshold for enucleation when response to globe-sparing therapy is inadequate or equivocal.

Strengths and limitations

The principal strength of this study lies in its focused clinicopathological correlation within a narrowly defined and clinically relevant subgroup of RB. By restricting analysis to Group D eyes and directly comparing primary and secondary enucleation, this study addresses a practical clinical dilemma that is frequently encountered but relatively underexplored in the pathological literature. All specimens were assessed using internationally accepted histopathological criteria, enhancing the validity and comparability of the findings.

Several limitations should be acknowledged. The small sample size, inherent to the rarity of enucleated Group D RB in the modern treatment era, limits statistical power and precludes definitive causal inference. The retrospective nature of the study introduces potential selection bias, as decisions regarding globe-sparing therapy and enucleation were made clinically rather than according to a standardized protocol. Furthermore, histopathological evaluation provides only a single time-point assessment, making it impossible to determine the temporal evolution of high-risk features. Despite these limitations, the consistency of the observed patterns with prior literature supports the relevance of the findings, particularly for clinicians working in resource-limited settings where treatment delays are more common.

Future research directions

The results of this study highlight clinically meaningful patterns that warrant further investigation. Prospective multicenter collaborations, particularly involving LMIC institutions, may help clarify optimal thresholds for transitioning from globe-sparing therapy to enucleation in advanced intraocular RB. Future studies incorporating molecular and genetic profiling may further elucidate tumor behavior and identify biological markers associated with treatment resistance and microscopic progression [21,22].

## Conclusions

In this clinicopathological analysis, high-risk histopathological features were more frequently identified in Group D RB eyes undergoing secondary enucleation following failed globe-sparing therapy compared with those treated by primary enucleation. Although limited by a small sample size, these findings suggest that delayed enucleation in selected advanced cases may permit progression of occult microscopic disease not evident on clinical examination. The results underscore the importance of vigilant assessment of treatment response and timely decision-making when managing advanced intraocular RB. In particular, early transition to enucleation should be considered for eyes demonstrating poor or incomplete response to conservative therapy, especially in settings where prolonged treatment courses or interruptions may increase oncologic risk. Further multicenter studies are needed to refine the criteria for enucleation timing and to optimize safe treatment strategies for Group D RB.
